# Validation of magnetic resonance imaging for quantification of intrapancreatic fat deposition using phantom and histologic comparators: a systematic review and meta-analysis

**DOI:** 10.1007/s00330-026-12475-x

**Published:** 2026-03-23

**Authors:** Jasmine Zhang, Yutong Liu, Maxim S. Petrov

**Affiliations:** School of Medicine, University of Auckland, Auckland, New Zealand

**Keywords:** Magnetic resonance imaging, Body fat distribution, Diabetes mellitus, Pancreatic neoplasms, Pancreatitis

## Abstract

**Objectives:**

Recent insights into understanding intrapancreatic fat deposition (IPFD) and its relationship with common pancreatic diseases have opened new opportunities for their prevention and treatment. This progress underscores the need for an accurate and universally applicable non-invasive imaging modality tailored for IPFD quantification. Chemical shift-encoded magnetic resonance imaging (CSE-MRI) is a non-ionising, time-efficient technique thought to be naturally fit for IPFD quantification because of its excellent fat–water separation. However, this assumption has not yet been systematically evaluated. The aim of this study was to conduct a systematic review assessing the validity of CSE-MRI in measuring IPFD.

**Materials and methods:**

A systematic search was performed in MEDLINE, Embase, and Scopus databases. Data from studies comparing CSE-MRI–derived measurements with known phantom fat values or histologically measured fat in the human pancreas were pooled using the Hedges-Olkin method.

**Results:**

A total of 13 studies were included. CSE-MRI-derived fat fraction (FF) demonstrated an excellent correlation with known FF in phantoms (*r* = 0.996; 95% CI: 0.992–0.998; *p* < 0.0001) and a strong correlation with histologically measured FF (*r* = 0.775; 95% CI: 0.675–0.847; *p* < 0.0001).

**Conclusion:**

These findings support the suitability of CSE-MRI for quantifying IPFD. Future research should focus on developing optimised and universally applicable imaging protocols.

**Key Points:**

***Question*** *Could chemical shift-encoded magnetic resonance imaging (CSE-MRI) become the modality of choice for non-invasive quantification of intrapancreatic fat deposition?*

***Findings**** Fat fraction measured by CSE-MRI closely matched known phantom values and correlated strongly with histologically determined fat in the pancreas*.

***Clinical relevance**** Broad use of CSE-MRI for quantifying intrapancreatic fat deposition has the potential to reduce the global burden of pancreatitis, pancreatic cancer, and type 2 diabetes mellitus*.

**Graphical Abstract:**

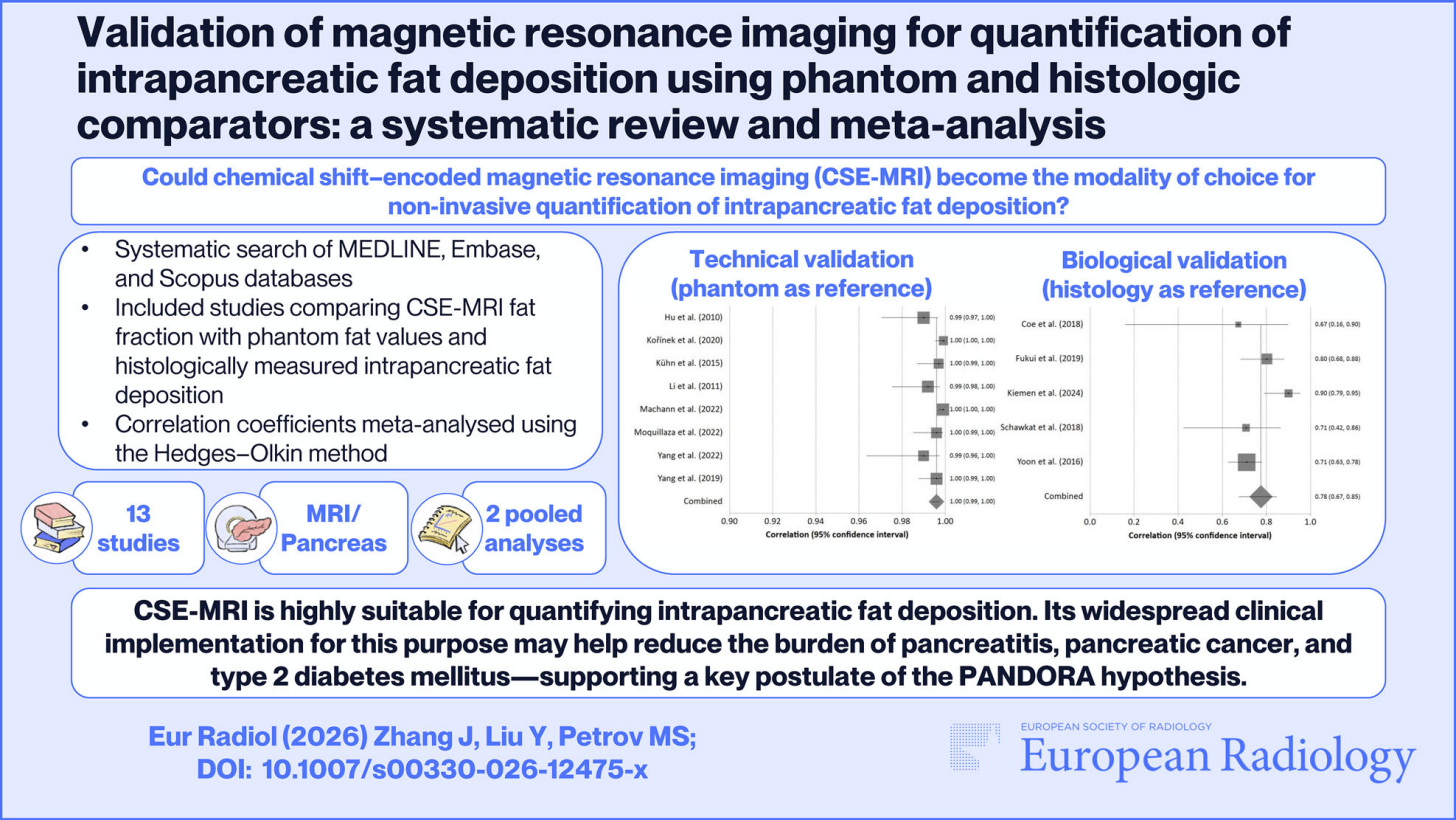

## Introduction

Intrapancreatic fat deposition (IPFD) is defined as the diffuse presence of fat within the pancreas, including intralobular and interlobular fat but excluding extralobular fat [1]. IPFD has recently been brought to the fore as research into lipotoxicity has expanded [1, 2]. While the healthy human pancreas contains minimal IPFD, excessive IPFD is strongly associated with an increased risk of pancreatic diseases [1, 2]. Moreover, the PANDORA (PANcreatic Diseases Originating from intRapancreatic fAt) hypothesis posits that excessive IPFD is a causal factor in the development of pancreatitis, pancreatic cancer, and type 2 diabetes mellitus (T2DM) [1–5]. Given the labile nature of IPFD, there is potential to reverse early disease states or even prevent the onset of common pancreatic diseases [1].

Invasive methods, such as endoscopic fine-needle biopsy and histological sampling during pancreatic surgery, can provide accurate measurements of IPFD but are not ethically justified for assessing excessive IPFD in most individuals [2]. Consequently, IPFD is predominantly measured using non-invasive imaging modalities [1]. Although magnetic resonance spectroscopy (MRS) is generally considered the gold standard for quantifying ectopic fat, it has notable limitations for IPFD, including long acquisition times, strict magnetic field homogeneity requirements, and a high risk of contamination from adjacent visceral fat [1, 6, 7]. Ultrasound is also less suitable due to its subjective measurement technique [1]. Computed tomography, while providing objective and (semi)quantitative data, is limited by the risks related to high ionising radiation exposure. Chemical shift–encoded magnetic resonance imaging (CSE-MRI) has been established as an accurate non-invasive method for assessing fat fraction (FF) in major organs other than the pancreas [1, 8]. However, the lack of validation for CSE-MRI for FF in the pancreas has limited its widespread adoption for quantifying IPFD. Previous original studies have used pancreatic histology and FF phantoms as reference comparators when evaluating the accuracy of CSE-MRI for measuring IPFD.

This study aimed to systematically review CSE-MRI studies that employed either fat phantoms or histological measurements of the human pancreas as reference standards.

## Materials and methods

### Search strategy

The literature search was conducted using three primary databases: MEDLINE, Embase, and Scopus. Separate strings were developed for phantom comparisons and histologic comparisons, with three strings used for each. All searches were conducted on April 25th, 2025, and the full search strings are provided in Supplementary Table 1. The authors’ personal libraries were also searched to identify any additional relevant studies.

### Eligibility criteria

Studies were eligible if they employed any chemical shift-based MRI technique to measure fat specifically within the pancreas. No restrictions were placed on MRI scanner manufacturers, magnetic field strength, geographic location, or language. Included studies needed to contain original data, involve a human population of ≥ 5, and report fat content in the pancreas as a fraction or percentage, allowing comparison with CSE-MRI–measured FF. Phantom comparators were eligible if they measured FF in any pancreas-specific imaging phantom, whereas histologic comparators were eligible if they measured FF in human pancreatic tissue. Case reports, conference proceedings, reviews, and study protocols were excluded. Studies were also excluded if the data could not be reconstructed to obtain a correlation coefficient (*r* or *r*^2^ value), if measured FF included any structures other than diffuse fat (as these are not considered IPFD [1]), or if data were collected across multiple scanners from different manufacturers.

### Methodological quality

The Joanna Briggs Institute’s critical appraisal tool was used to assess all eligible studies [9]. The instrument consists of eight questions evaluating key study features, with some questions adapted to better suit the studies under review. For example, the validity and reliability requirements were considered met in phantom comparator studies if appropriate phantom materials and construction methods were used and the constructed FF values were reported. In histologic comparator studies, this criterion was met if reasonable histology preparation and analysis methods were described. For criterion five, confounding factors were identified if the study reported potential CSE-MRI biases in measuring FF; criterion six addressed whether bias correction was applied. Questions were answered as “Yes”, “No”, or “Unclear” [9].

### Statistical analysis

Meta-correlation was used to assess the relationship between CSE-MRI-measured pancreatic FF and FF measured either by phantoms or histology. Data were analysed using the meta-analysis of correlations function in StatsDirect (version 3.2.7). The Hedges-Olkin meta-correlation method was applied to generate forest plots, and a random effects model was used to provide the most conservative estimate. For each study, correlation coefficients reported as *r*^2^ were converted to *r*-values [10]. Correlation coefficients were statistically pooled separately for studies using phantom and histologic comparators to obtain two pooled estimates of the strength of association. Correlations were interpreted as weak (*r* ≤ 0.3), moderate (0.3 < *r* < 0.7), or strong (*r* ≥ 0.7) [11].

The *Z*-test was used to assess the overall effect, with the null hypothesis that the mean effect size equals zero. The *I*^2^ statistic was used to evaluate statistical heterogeneity across studies. As *I*^2^ represents the percentage of total variation attributable to between-study heterogeneity rather than chance, values of < 25% were interpreted as low heterogeneity, 25%–75% as moderate heterogeneity, and > 75% as high heterogeneity. A *p*-value of < 0.05 was considered statistically significant. Publication bias was assessed using Egger’s test, which applies weighted linear regression of effect size estimates against their precision [12]. A statistically significant Egger’s test *p*-value was interpreted as evidence of potential publication bias [12].

## Results

### Study characteristics

**Fig. 1:**
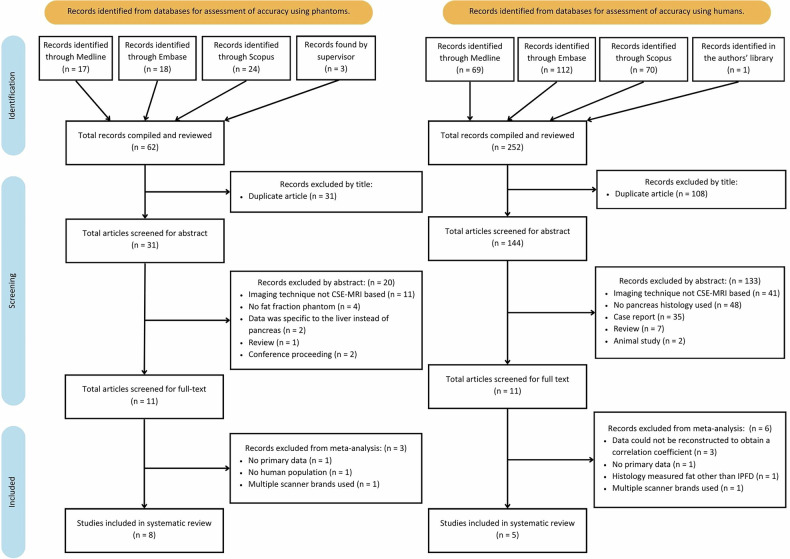
Study selection process

The results of the search and screening process are presented in Fig. 1. The search yielded eight studies using phantom comparators, published between 2010 and 2025 [6, 7, 13–18], which predominantly included healthy individuals from five different countries (Table 1). Five studies using histologic comparators were also identified, published between 2016 and 2024 [19–23]. These studies included patients with pancreatic diseases from five different countries (Table 1); two studies additionally included non–high-risk control participants undergoing pancreatic resection for benign lesions [20, 21]. The methodological quality of the included studies is summarised in Supplementary Table 2.

**Table 1:** Characteristics of all included studies

Study ID	Country	Year	Study design	Comparator	Participant characteristics	No. of participants (M:F)	Age (years)
Coe et al [19]	UK	2018	Preoperative pancreatic FF was determined through MRS and CSE-MRI; MRI-derived pancreatic FF was compared with histological digital fat quantification.	Histology	Patients undergoing pancreatic resection	12 (10:2)	45–82^a^
Fukui et al [20]	Japan	2019	Histological pancreatic FF was measured from non-tumorous tissue and compared with preoperative MRI-PDFF.	Histology	Patients undergoing pancreatectomy, without receiving preoperative therapy: pancreatic cancer (*n* = 24), controls without pancreatic cancer (*n* = 31)	55	72^b^
Hu et al [13]	USA	2010	A phantom was constructed to assess the accuracy of IDEAL MRI in evaluating FF.	Phantom	Healthy	16 (7:9)	24–54^a^
Kiemen et al [21]	USA	2024	MRI was used to estimate pancreatic FF in preoperative images, which was then correlated with histological tissue composition.	Histology	High pancreatic cancer risk participants under regular surveillance who underwent pancreatic resection (*n* = 15). Non–high-risk control individuals who underwent surgical resection for benign pancreatic lesions or nonpancreatic lesions (*n* = 12)	27 (12:15)	64.0 ± 12.0^c^
Kořínek et al [14]	Austria	2020	A small phantom was processed using three different software packages to assess the accuracy of PDFF quantification, whereas a large phantom was used to evaluate the effects of substantial B_0_ field inhomogeneity. FF measured by MRI-Dixon at various levels was compared against both MRS measurements and the expected FF values.	Phantom	Healthy	7 (3:4)	29–35^a^
Kühn et al [15]	Germany	2015	A phantom was used to investigate the validity of the PDFF approach for quantifying tissue fat in the pancreas.	Phantom	Healthy	1367 (563:678)	45–60^a^
Li et al [16]	China	2011	A phantom was used to establish the accuracy of the MRI sequence.	Phantom	Healthy	126 (126:0)	20–70^a^
Machann et al [17]	Germany	2022	A phantom was constructed to evaluate the feasibility of CSE-MRI for detecting small changes in FF.	Phantom	Healthy	Part 1: 12 (6:6) Part 2: 15 (15:0) Part 2 Controls: 11 (11:0)	Part 1: 24–49^a^ Part 2: 19–27^a^ Part 2 Controls: 20–26^a^
Moquillaza et al [18]	Germany	2025	MRI-PDFF was used to confirm the FF of the phantoms, which were then used to evaluate the accuracy of the proposed wT1 method relative to T1-MOLLI and MRS.	Phantom	Healthy (*n* = 11), necrotising pancreatitis (*n* = 4)	15	Not reported
Schawkat et al [22]	Switzerland	2018	Preoperative MRI measurements were compared with histologically determined pancreatic FF.	Histology	Patients undergoing pancreatectomy	24 (16:8)	68–74^a^
Yang et al [6]	New Zealand	2022	The known FF of the phantom was compared with FF measurements obtained using MRS, IDEAL MRI, and the MR-opsy method.	Phantom	Healthy	16 (6:10)	20–55^a^
Yang et al [7]	China	2019	A phantom was employed to construct a linear regression calibration curve, which was then used to estimate the true FF.	Phantom	Healthy	167 (0:167)	20–70^a^
Yoon et al [23]	South Korea	2016	FF estimated from preoperative MR images was compared with histologically measured FF from surgical specimens.	Histology	Patients undergoing pancreatectomy	165 (93:72)	62^c^

*FF* fat fraction, *IDEAL* iterative decomposition of water and fat with echo asymmetry and least-squares estimation, *PDFF* proton density fat fraction, *MRS* magnetic resonance spectroscopy

In the study by Machann et al [17], Part 1 participants were tested for intraday changes in pancreatic FF, whereas Part 2 participants underwent a high-caloric diet for five days to assess for medium-term changes in pancreatic FF

^a^ Range

^b^ Median

^c^ Mean ± SD

### CSE-MRI protocols

A summary of the CSE-MRI parameters and acquisition specifications for studies using phantom comparators is presented in Table 2. All phantom studies employed a 3-T magnet, except one, which used a 1.5-T system [15]. Two studies employed a double-echo technique [7, 16], whereas six studies used multi-echo techniques [6, 13–15, 17, 18]. Repetition times (TR) ranged from 8.6 to 150 ms, whereas echo spacing ranged from 0.2 to 4.8 ms. Flip angles (FAs) ranged from 3° to 85°. All studies, except one [13], used a gradient-echo acquisition. A summary of the CSE-MRI parameters and acquisition specifications for studies using histologic comparators is presented in Table 3. All studies employed a 3-T magnet, except one that used a 1.5-T system [19]. Only one study used a dual-echo technique [21], whereas the remaining studies employed multi-echo acquisitions; TE data were unavailable for one study [20]. TR ranged from 3.8 to 150 ms, and echo spacing ranged from 1.2 to 4.6 ms. FA ranged from 3° to 11°. Three studies reported the use of gradient-echo sequences [19, 22, 23]. Only the studies by Fukui et al [20] and Kiemen et al [21] reported the interval between MRI acquisition and specimen collection, with median intervals of 50 and 56 days, respectively. Four studies reported the use of multiple readers for CSE-MRI–based assessment of pancreatic FF [6, 17, 19, 21]. Additional measurement details are provided in Supplementary Table 3.

**Table 2:** CSE-MRI techniques in studies using phantom comparators

Study ID	Scanner			CSE-MRI sequence for FF quantification	Parameters						Additional bias correction
	Manufacturer	Strength (T)	Model		TR (ms)	TE (ms)	Echo spacing (ms)	FA (degrees)	Section thickness (mm)	Matrix size (px)	
Hu et al [13]	GE HealthCare	3	Signa HDx	3D IDEAL	10	2.0, 2.4, 2.8, 3.2, 3.6, 4.0	0.4	5	5	160 × 160	None.
Kořínek et al [14]	Siemens Healthineers	3	Siemens Trio	3D spoiled gradient recalled (SPQR) sequence	9.32	1.23, 2.54, 3.85, 5.16, 6.47, 7.78	1.31	3	Not reported	160 × 160	T1 effect minimised with small flip angle. T2* correction: T2* estimate included in VARPRO assessment.
Kühn et al [15]	Siemens Healthineers	1.5	MAGNETOM Avanto	3D gradient-echo chemical shift-encoded pulse sequence	11	2.4, 4.8, 9.6	2.4, 4.8	10	Not reported	224 × 168	T1 bias, T2* bias, multipeak spectral complexity of fat, and noise bias correction.
Li et al [16]	GE HealthCare	3	Signa Excite	2D double-echo chemical shift gradient-echo sequence	150	2.5, 5.8	3.3	85	Not reported	Not reported	T1/T2* correction. Independent T2* sequence to estimate the fat/water values and correct for it.
Machann et al [17]	Siemens Healthineers	3	MAGNETOM Vida	3D multi-echo gradient-echo sequence	20	1.09, 2.46, 3.69, 4.92, 6.15, 7.38	1.37, 1.23, 1.23, 1.23, 1.23	4	3	160 × 132	T2* correction.
Moquillaza et al [18]	Philips Healthcare	3	Ingenia Elition X	3D Dixon spoiled gradient-echo sequence	8.6	1.19, 2.39, 3.59, 4.79, 5.99, 7.19	1.2	3	5	225 × 225	B_1_ correction accounted for T1 being biased in the presence of fat.
Yang et al [6]	Siemens Healthineers	3	MAGNETOM Skyra	3D IDEAL spoiled gradient-echo sequence	10	2, 4, 6, 8, 3.2, 3.6, 4.0	2.0, 0.2, 0.4	5	8	Not reported	Fat spectrum model was used to account for overestimation of T2* value.
Yang et al [7]	Siemens Healthineers	3	MAGNETOM Skyra	2D spoiled double-echo gradient-echo sequence	80	1.23, 2.46	1.23	50	5	352 × 286	T1/T2* correction. Independent sequence used to estimate equal T2* of fat and water.

*FF* fat fraction, *TR* repetition time, *TE* echo time, *FA* flip angle, *IDEAL* iterative decomposition of water and fat with echo asymmetry and least-squares estimation

**Table 3:** CSE-MRI techniques in studies using histologic comparators

Study ID	Scanner			CSE-MRI sequence for FF quantification	Parameters						Additional bias correction
	Manufacturer	Strength (T)	Model		TR (ms)	TE (ms)	Echo spacing (ms)	FA (degrees)	Section thickness (mm)	Matrix size (px)	
Coe et al [19]	Philips Healthcare	1.5	Achieva	Multigradient-echo Dixon	150	2.3, 4.6, 9.2	2.3, 4.6	Not reported	50	128 × 96	T2*-decay correction: IP signal calculated using acquisitions at 4.6 ms & 9.2 ms.
Fukui et al [20]	GE HealthCare	3	Discovery MR750/MR750w	IDEAL-IQ	5.8 or 6.7	Not reported	Not reported	3	8	128 × 128	None.
Kiemen et al [21]	Siemens Healthineers	3	MAGNETOM Tim Trio	3D dual-echo Dixon	3.8	1.22, 2.45	1.23	10	3	Not reported	None.
Schawkat et al [22]	Siemens Healthineers	3	Skyra	Multigradient-echo Dixon	9	1.05, 2.46, 3.69, 4.92, 6.15, 7.38	1.41, 1.23, 1.23, 1.23, 1.23	4	3.5	160 × 140	T2*-decay was corrected via multigradient echo.
Yoon et al [23]	Siemens Healthineers	3	MAGNETOM Verio	3D gradient-echo Dixon	9.89	2.45, 3.67, 7.35	1.22, 3.68	11	4	256 × 167	T2* correction: voxel-wise correction applied by using T2* values calculated from signal intensity and echo times of two IP images with linear fit in log space.

*FF* fat fraction,* IP* in-phase, *TR* repetition time, *TE* echo time, *FA* flip angle, *IDEAL* iterative decomposition of water and fat with echo asymmetry and least-squares estimation

### Phantom and histology protocols

All phantoms were vial-based and consisted of either vegetable oil or Intralipid emulsion diluted with distilled water, with each phantom comprising multiple vials (8–16 vials). Four studies used larger FF increments of at least 10% [6, 13, 15, 17], whereas the remaining studies employed smaller increments ranging from 0.2% to 1.12% [7, 14, 16–18]. All studies using histologic comparators employed haematoxylin and eosin (H&E) staining; detailed histological methods are summarised in Supplementary Table 4. Histology-determined FF ranged from 0% to 70% whereas CSE-MRI–measured FF ranged from 0% to 39.1%. In the two histologic comparator studies that included multiple readers, the mean FF values across readers were used for analysis [19, 21]. Of the four studies that reported the site of tissue sampling, specimens were consistently obtained from the pancreatic resection margin to ensure consistency and ease of access [19, 20, 22, 23]; however, the study by Schawkat et al [22] also collected samples from the pancreatic tail. The number of histological slides acquired per participant was reported in only two studies: Coe et al [19] collected eight slides per participant, whereas Kiemen et al [21] collected a median of 12 slides per participant.

### Validation of CSE-MRI for quantifying IPFD

**Fig. 2:**
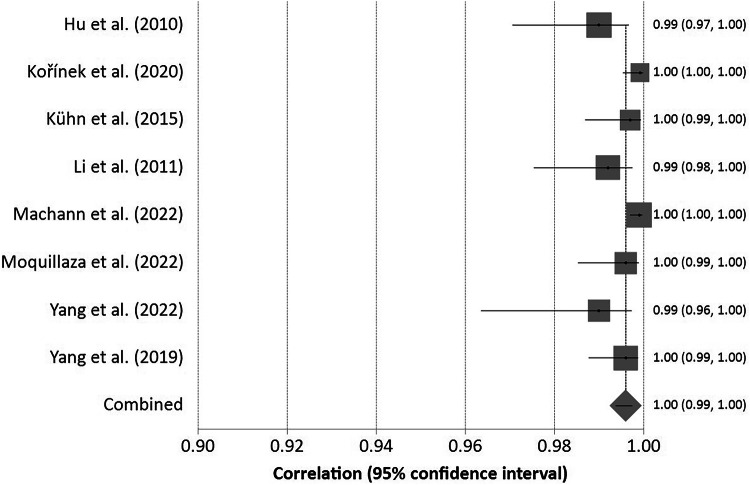
Validity of CSE-MRI in studies using phantom comparators. Multiple regression values were reported in the study by Kořínek et al [14]. For the meta-analysis, the regression value obtained from the FatWater12/Graph-Cut software trial was selected, as it was determined to be the most robust (exhibiting the fewest water–fat swaps in the PDFF maps). In the forest plot, the size of each cube represents the effect size of an individual study, whereas the diamond indicates the overall pooled effect size of the combined correlation values

**Fig. 3:**
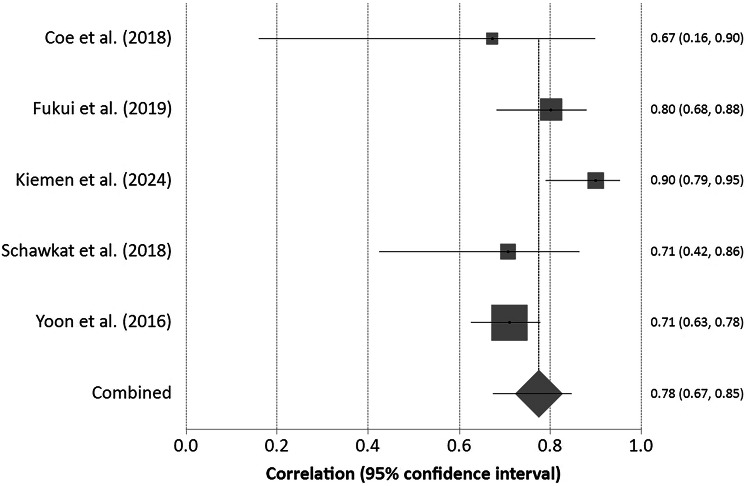
Validity of CSE-MRI in studies using histologic comparators. Multiple regression values were reported in the study by Schawkat et al [22]. For the meta-analysis, the regression value from the pancreatic tail was selected, as this site is more generalisable to the broader population compared with the resection margin. In the forest plot, the size of each cube represents the effect size of an individual study, whereas the diamond indicates the overall pooled effect size of the combined correlation values

CSE-MRI–measured FF was strongly positively correlated with the known FF of phantoms (*r* = 0.996; 95% CI: 0.992–0.998; *p* < 0.0001) (Fig. 2). Statistical heterogeneity was moderate, with *I*² = 55.5% (95% CI: 0%–78%). Egger’s regression test indicated a low likelihood of publication bias in the phantom comparator studies (score = 2.95; 95% CI: −6.07–11.98; *p* = 0.454). Similarly, CSE-MRI–measured FF was positively correlated with FF measured using histological slides (*r *= 0.775; 95% CI: 0.675–0.847; *p* < 0.0001) (Fig. 3). Heterogeneity was moderate (*I*² = 52.7%; 95% CI: 0%–80.7%), and Egger’s regression test suggested a low likelihood of publication bias in the histologic comparator studies (score = 1.13; 95% CI: −3.59–5.84; *p* = 0.502).

## Discussion

This systematic review and meta-analysis is the first to validate the use of CSE-MRI for IPFD quantification. A total of 13 CSE-MRI studies were included, comprising eight studies using phantom comparators and five using histologic comparators, and encompassing over 2000 participants. The studies included in this systematic review were generally of high methodological quality, and the risk of publication bias was low. The analysed data consisted of correlation coefficients between CSE-MRI–derived FF measurements and known phantom FF values, as well as correlations between pancreatic FF measured by CSE-MRI and by histology.

The pooled results from the phantom studies demonstrated a correlation of 0.996 between CSE-MRI–measured pancreatic FF and the known fat values of the phantoms, indicating near-perfect accuracy under controlled conditions. This novel finding supports the hypothesis that CSE-MRI is a highly sensitive method for quantifying IPFD [1]. The robustness of this result is further reinforced by the consistency of phantom measurements and the reproducibility of phantom construction. The pooled results from the histology studies demonstrated a strong correlation of 0.775 between CSE-MRI–measured pancreatic FF and histologically sampled FF. Although the correlation was not perfect, the observed level of accuracy is considered sufficient for clinical application in the quantification of IPFD [1, 16, 24]. The less-than-perfect correlation may be attributable to limitations inherent in histologic methods employed in the included studies. Notably, all five studies used H&E staining, which is optimised for identifying adipocytes but cannot precisely characterise lipid content within other pancreatic cell types that also contribute to IPFD [25]. The H&E staining method was effective in detecting only a portion of intralobular fat—specifically intralobular adipocytes—while lipid droplets within endocrine and acinar cells were not fully represented [1, 25]. Similarly, only a portion of interlobular fat (namely, interlobular adipocytes) was captured, whereas lipid droplets in quiescent stellate cells were detected less accurately [1]. Consequently, because H&E staining is nonspecific for the quantification of IPFD, the reported histologic measurements likely underestimate the true pancreatic FF. The extent of unmeasured fat cannot be quantified, as no regression equations were provided to account for this discrepancy. An improved approach, as demonstrated by Luchini et al [25], incorporates additional Oil Red O staining—a well-established and highly sensitive histochemical technique for detecting lipid droplets.

Several additional factors may have contributed to the observed phantom–human gap. Although careful region of interest (ROI) placement avoided major blood vessels and the main pancreatic duct, the presence of blood flow in vivo likely alters the dielectric properties of pancreatic parenchyma compared with histologic specimens. In addition, sampling mismatch may arise from the limited number of histologic sections per case—most studies sampled only the resection margin—as well as from delays between imaging and histologic analysis, which may introduce variability in parenchymal composition or sampling location. Two studies reported median MRI-to-histology delays of 50 and 56 days [20, 21], and it remains uncertain whether these intervals are associated with notable changes in pancreatic parenchyma (including IPFD or fibrosis) [26]. Respiratory motion during in vivo imaging further contributes to potential mismatches in ROI localisation. Improved co-localisation of imaging ROIs with standardised histologic sampling across the pancreatic head, body, and tail—as implemented in some CSE-MRI protocols—may better capture true correlation between CSE-MRI–measured pancreatic FF and histologically sampled FF. Collectively, unavoidable differences between histologic and in vivo conditions, together with the relatively crude histologic methods employed in the included studies, likely contributed to the attenuated pooled correlation observed. Nevertheless, these limitations do not undermine the clinical utility of CSE-MRI for quantifying IPFD [27].

The clinical applications of the studies reviewed were pertinent primarily to the first element of the PANDORA hypothesis [5]. CSE-MRI is well-suited for pancreatic FF quantification clinically for its speed, accuracy, lower magnetic field homogeneity requirements compared with MRS, and safety for patients; it is non-invasive and does not utilise ionising radiation [1]. Therefore, early morphological changes of the pancreas that are not primarily genetic in origin can be identified using CSE-MRI [5, 28–30]. CSE-MRI was found to be useful in identifying baseline limits in healthy populations for IPFD, in the investigation of its correlation with other clinical symptoms (from physical measurements to fibrosis and disease states), and as a precursor of pancreatic diseases such as pancreatic cancer [19–21]. The use of CSE-MRI as the modality of choice for quantifying IPFD will therefore facilitate research into the identification and prevention of pancreatic diseases, supporting progress towards reducing the burden of pancreatitis, T2DM, and pancreatic cancer worldwide [1, 31]. A standardised CSE-MRI protocol for measuring IPFD has not yet been established, and the effects of specific imaging parameters on IPFD quantification remain under-investigated. Technically, CSE-MRI is relatively unaffected by magnetic field strength inhomogeneity and performs well across a range of B_0_ field strengths [16]. Three studies across this systematic review used the IDEAL technique, which adequately corrects for T1- and T2*-biases [6, 13, 20, 32]. In Dixon sequences, T2*-decay was corrected via calculation of T2* values from IP (in-phase) images, while T1-bias was corrected using literature values of the T1 relaxation times for fat and water [6, 7, 16, 19, 22, 23, 33]. These techniques were accurate and feasible in clinical application, though one drawback of the IDEAL sequence is that it can only be used on certain MRI scanners with the specific sequence [7]. A conventional technique of correcting T1-bias employed across several studies is a long TR and small FA, either in conjunction or separately [34]. A long TR is not ideal in clinical application due to the increased scanning time and therefore breath-hold requirement, increasing patient motion artefact; a smaller FA also lowers the signal-to-noise ratio and has been shown to lead to the false detection of fat in the spleen [33]. A future research direction would be the possibility of standardised correction techniques for CSE-MRI in IPFD quantification. A potential future research direction is the development of standardised correction techniques for CSE-MRI in the quantification of IPFD.

Another aspect of the clinical application of CSE-MRI for measuring IPFD relates to factors affecting measurement precision, including technical repeatability, inter-reader reproducibility, and consistency in ROI selection. Macchan et al [17] reported negligible short-term changes (same-day and up to five days) in pancreatic FF in human participants, even after a high-calorie diet, supporting the short-term technical repeatability of CSE-MRI. As only two studies in the present review reported medium-term imaging-to-histology delays [20, 21], further analysis of technical repeatability was not feasible. ROI selection was typically based on individual pancreas size, with the largest feasible area used while avoiding the main pancreatic duct, vessels, and visceral adipose tissue (range: 0.4–206.8 mm²). Several studies employed a modified ‘MR-opsy’ approach, placing ROIs in the pancreatic head, body, and tail [6, 7, 15–19], and averaging pancreatic FF across multiple axial slices to improve accuracy [35–40]. Only two primary studies assessed inter-reader variability, both reporting low variation [19, 21]. Coe et al [19] found a correlation of 0.91 for inter-rater variability, whereas Kiemen et al [21] reported a median percent difference of 1% between two observers’ FF estimates. Although additional data on factors influencing repeatability and reproducibility would further strengthen the clinical utility of CSE-MRI for quantifying IPFD, this was beyond the primary focus of the present review and the search strategy was not specifically designed to capture studies addressing these aspects.

Several limitations of this systematic review should be acknowledged. First, the relatively small number of included studies may reduce the reliability of pooled estimates. Moderate heterogeneity was observed in both phantom comparator studies (*I*² = 55.5%) and histologic comparator studies (*I*² =  52.7%), at least in part due to substantial variation in MRI parameters such as TR (3.8–150 ms) and FA (3–85°). Further investigation of heterogeneity through subgroup or sensitivity analyses was not feasible due to the limited number of studies. Nevertheless, this is the first systematic review on this topic and provides a foundation for future imaging studies on IPFD. Second, the primary statistical metric used—the correlation coefficient—may be considered a surrogate for the accuracy of CSE-MRI–derived IPFD quantification. Although agreement-based metrics, such as Bland-Altman analysis, could potentially strengthen the findings, individual patient data were not available to enable such analyses. Future investigations should consider the use of agreement-based metrics. Third, the fat phantoms have inherent limitations. Unlike human tissue, phantoms are not affected by respiratory motion, body temperature, pancreatic tissue heterogeneity, or blood flow. However, these limitations were unavoidable and do not detract from the high reliability of the phantom measurements. Last, histologic comparator studies were restricted to individuals undergoing pancreatic surgery, typically for pancreatic cancer, which may limit generalisability to the broader population. This limitation is partially mitigated by the fact that all studies sampled non-tumourous pancreatic parenchyma, as obtaining pancreatic tissue from healthy participants would be unethical.

In conclusion, quantifying IPFD using CSE-MRI represents a novel clinical approach with the potential to improve early detection and management of pancreatic diseases. CSE-MRI shows high comparability with both phantom models and histologic measurements, and is safe, versatile, and feasible for routine use. It provides valuable insights into IPFD and its associations with pancreatic disorders. Widespread implementation of CSE-MRI for IPFD quantification is therefore warranted to help reduce the burden of pancreatitis, pancreatic cancer, and T2DM.

## Supplementary information

ELECTRONIC SUPPLEMENTARY MATERIAL

## Abbreviations

CSE-MRI: Chemical shift-encoded magnetic resonance imaging
FA: Flip angle
FF: Fat fraction
H&E: Haematoxylin & eosin
IDEAL: Iterative decomposition of water and fat with echo asymmetry and least-squares estimation
IP: In-phase
IPFD: Intrapancreatic fat deposition
MRI: Magnetic resonance imaging
MRS: Magnetic resonance spectroscopy
PANDORA: PANcreatic Diseases Originating from intRa-pancreatic fAt
PDFF: Proton density fat fraction
ROI: Region of interest
T2DM: Type 2 diabetes mellitus
TE: Echo time
TR: Repetition time
