# Supplementary material for: Validation of magnetic resonance imaging for quantification of intrapancreatic fat deposition using phantom and histologic comparators: a systematic review and meta-analysis
Source: Eur Radiol. 2026 Mar 23;36(8):6546–58. doi: 10.1007/s00330-026-12475-x (PMC13341726; doi:10.1007/s00330-026-12475-x)
Supplement: Supplementary file 1 — ELECTRONIC SUPPLEMENTARY MATERIAL [file 330_2026_12475_MOESM1_ESM.pdf]

Validation of Magnetic Resonance Imaging for Quantification of Intra-Pancreatic Fat Deposition Using Phantom and Histologic Comparators: A Systematic Review and Meta-analysis

ELECTRONIC SUPPLEMENTARY MATERIAL

Supplementary Table 1. Strings used for the literature search.

Database	Phantom comparator studies	Histologic comparator studies
MEDLINE	Pancreas/ or pancrea*.ti,ab,kw. AND Magnetic Resonance Imaging/ or (chemical shift-encoded MRI or CSE-MRI or CS-MRI or CSI or "chemical shift-encoded magnetic resonance imaging" or "chemical shift encoded magnetic resonance imaging" or "chemical shift magnetic resonance imaging" or "chemical shift-encoded MRI" or "chemical shift MRI" or MRI or "magnetic resonance imaging").ti,ab,kw. AND (intra-pancreatic fat deposition or IPFD or "intrapancreatic fat deposition" or "pancreatic fat fraction" or "pancreatic fat" or steatosis or lipid or fat or "fat deposition" or lipomatosis).ti,ab,kw. AND Phantoms, Imaging/ or (phantom or "imaging phantom").ti,ab,kw.	Pancreas/ or pancrea*.ti,ab,kw. AND Magnetic Resonance Imaging/ or (chemical shift-encoded MRI or CSE-MRI or CS-MRI or CSI or "chemical shift-encoded magnetic resonance imaging" or "chemical shift encoded magnetic resonance imaging" or "chemical shift magnetic resonance imaging" or "chemical shift-encoded MRI" or "chemical shift MRI" or MRI or "magnetic resonance imaging").ti,ab,kw. AND (intra-pancreatic fat deposition or IPFD or "intrapancreatic fat deposition" or "pancreatic fat fraction" or "pancreatic fat" or steatosis or lipid or fat or "fat deposition" or "lipomatosis").ti,ab,kw. AND Biopsy/ or biopsy.ti,ab,kw. or Autopsy/ or autopsy.ti,ab,kw. or Histology/ or histolog*.ti,ab,kw. or pancrea* biopsy.ti,ab,kw. limit to (humans and (classical article or comparative study or "corrected and republished article" or dataset or journal article or multicenter study or observational study))
Embase	pancreas/ or pancrea*.ti,ab,kw. AND nuclear magnetic resonance imaging/ or (chemical shift-encoded MRI or CSE-MRI or CS-MRI or CSI or "chemical shift-encoded magnetic resonance imaging" or "chemical shift encoded magnetic resonance imaging" or "chemical shift magnetic resonance imaging" or "chemical shift-encoded MRI" or "chemical shift MRI" or MRI or "magnetic resonance imaging").ti,ab,kw. AND lipid storage/ or ("pancreatic fat" or "intra-pancreatic fat	pancreas/ or pancrea*.ti,ab,kw. AND nuclear magnetic resonance imaging/ or (chemical shift-encoded MRI or CSE-MRI or CS-MRI or CSI or "chemical shift-encoded magnetic resonance imaging" or "chemical shift encoded magnetic resonance imaging" or "chemical shift magnetic resonance imaging" or "chemical shift-encoded MRI" or "chemical shift MRI" or MRI or "magnetic resonance imaging").ti,ab,kw. AND lipid storage/ or ("pancreatic fat" or "intra-pancreatic fat

	deposition" or IPFD or "intrapancreatic fat deposition" or "pancreatic fat fraction" or "pancreatic fat" or steatosis or lipid or fat or "fat deposition" or “lipomatosis”).ti,ab,kw. AND imaging phantom/ or ("imaging phantom" or phantom or "anthropomorphic phantom").ti,ab,kw.	deposition" or IPFD or "intrapancreatic fat deposition" or "pancreatic fat fraction" or "pancreatic fat" or steatosis or lipid or fat or "fat deposition" or "lipomatosis").ti,ab,kw. AND biopsy/ or pancreas biopsy/ or biopsy.ti,ab,kw. or histology/ or histolog*.ti,ab,kw. or autopsy.ti,ab,kw. or autopsy/ or minimally invasive autopsy/ or clinical autopsy/ limit to (human and (article or article in press or data paper))
Scopus	(magnetic AND resonance AND imaging) OR (TITLE-ABS-KEY (mri)) OR (TITLE-ABS-KEY (chemical AND shift AND magnetic AND resonance AND imaging)) OR (TITLE-ABS-KEY (chemical AND shift AND encoded AND mri)) OR (TITLE-ABS-KEY (chemical AND shift AND encoded AND magnetic AND resonance AND imaging)) OR (TITLE-ABS-KEY (cse-mri)) OR (TITLE-ABS-KEY (chemical AND shift-encoded AND magnetic AND resonance AND imaging)) OR (TITLE-ABS-KEY (cs-mri)) OR (TITLE-ABS-KEY (csi)) AND (TITLE-ABS-KEY (pancrea*)) AND (TITLE-ABS-KEY (fat)) OR (TITLE-ABS-KEY (intrapancreatic AND fat AND deposition)) OR (TITLE-ABS-KEY (steatosis)) OR (TITLE-ABS-KEY(lipomatosis)) OR (TITLE-ABS-KEY (fat AND deposition)) OR (TITLE-ABS-KEY (lipid)) AND (TITLE-ABS-KEY (phantom)) OR (TITLE-ABS-KEY (imaging AND phantom))	(magnetic AND resonance AND imaging) OR (TITLE-ABS-KEY (mri)) OR (TITLE-ABS-KEY (chemical AND shift AND magnetic AND resonance AND imaging)) OR (TITLE-ABS-KEY (chemical AND shift AND encoded AND mri)) OR (TITLE-ABS-KEY (chemical AND shift AND encoded AND magnetic AND resonance AND imaging)) OR (TITLE-ABS-KEY (cse-mri)) OR (TITLE-ABS-KEY (chemical AND shift-encoded AND magnetic AND resonance AND imaging)) OR (TITLE-ABS-KEY (cs-mri)) OR (TITLE-ABS-KEY (csi)) AND (TITLE-ABS-KEY (pancrea*)) AND (TITLE-ABS-KEY (intrapancreatic AND fat AND deposition)) OR (TITLE-ABS-KEY (steatosis)) OR (TITLE-ABS-KEY(LIPOMATOSIS)) OR (TITLE-ABS-KEY (fat AND deposition)) AND (TITLE-ABS-KEY (histolog*)) OR (TITLE-ABS-KEY (autopsy)) OR (TITLE-ABS-KEY(tissue AND sample)) OR (TITLE-ABS-KEY(biopsy)) AND NOT animal SRCTYPE(j) AND NOT ultrasound AND (LIMIT-TO (DOCTYPE,"ar"))

Supplementary Table 2. Methodological quality assessment of the included studies.

**Study ID**	**Joanna Briggs Institute appraisal score (cross-sectional studies)**							
	**1**	**2**	**3**	**4**	**5**	**6**	**7**	**8**
Coe et al. [1]	Y	Y	Y	Y	Y	Y	U	Y
Fukui et al. [2]	Y	Y	Y	Y	N	N	U	Y
Hu et al. [3]	Y	Y	Y	Y	N	N	Y	Y
Kiemen et al. [4]	Y	Y	Y	Y	N	N	Y	Y
Kořínek et al. [5]	Y	Y	Y	Y	Y	Y	Y	Y
Kühn et al. [6]	Y	Y	Y	Y	Y	Y	Y	Y
Li et al. [7]	Y	Y	Y	Y	Y	Y	Y	Y
Machann et al. [8]	Y	Y	Y	Y	Y	N	Y	Y
Moquillaza et al. [9]	Y	Y	Y	Y	Y	Y	Y	Y
Schawkat et al. [10]	Y	Y	Y	Y	Y	Y	Y	Y
Yang et al. [11]	Y	Y	Y	Y	Y	Y	Y	Y
Yang et al. [12]	Y	Y	Y	Y	Y	Y	Y	Y
Yoon et al. [13]	Y	Y	Y	Y	Y	Y	Y	Y

Abbreviations: Y, yes; N, no; U, unclear.

Supplementary Table 3. Technical factors influencing CSE-MRI measurement of pancreatic fat fraction in human participants.

Study ID	Workstation	Measurement of pancreatic FF	Inter-reader variability
Coe et al. [1]	OsiriX	ROI size: determined by individual pancreas size.<br>Location: pre-treatment anticipated resection margin (determined using SMV/SV confluence and SMA). ROI placed at the head, body and tail. The average SI was taken.<br>FF = (IP-OP) / 2IP	Number of readers not reported.<br>r = 0.91 for inter-rater variability.<br>CV for the measurement of pancreatic head, body and tail were 33%, 31% and 36% respectively.
Fukui et al. [2]	Synapase; Fujifilm	ROI size: largest possible ellipse.<br>Location: estimated transection line (confirmed with surgeon or using operation records).	Single reader assessment.
Hu et al. [3]	Investigational IDEAL software	ROI size: not reported for CSE-MRI.<br>Location: not reported.<br>IDEAL = F / (W+F)	Number of readers not reported.
Kiemen et al. [4]	Not reported	ROI size: at least 1 cm^2<br>Location: first placed on IP image, then copy/paste to OP to ensure same position. FF taken at site of resection.<br>FF = (IP-OP) / 2IP	Two readers (blind) and average between the two readers was used.<br>The percent difference in the two observer's fat fraction estimation was a median of 1% (interquartile range, 2%).<br>The median overlap in manual annotations excluding nonpancreatic structures was 89.9% (interquartile range, 12.7%).
Kořínek et al. [5]	MATLAB (The MathWorks)	Not reported.	Number of readers not reported.
Kühn et al. [6]	OsiriX	ROI size: adjusted to the largest size possible to match the size of the pancreas.<br>Location: one ROI each at the head, body, and tail of the pancreas	Single reader assessment.
Li et al. [7]	GE system workstation and vendor software package	ROI size: 0.4-0.6cm^2<br>Location: at the head, body and tail of the pancreas, 3 (IP) and 6 (OP) ROI were placed over 3 adjacent layers. The mean signal intensity values for the ROI were recorded by the software package.<br>FF = (IP - OP) / 2IP	Single reader assessment.

Machann et al. [8]	Not reported	ROI size: not reported.<br>Location: three ROI at the head, body and tail of the pancreas (different axial slices where possible). The mean value of the three ROI's was calculated	Two readers.
Moquillaza et al. [9]	Vendor software	ROI size: circular and varying from 6.5 to 17.4 mm according to the anatomy.<br>Location: the head, body and tail of the pancreas. The average was taken.	Number of readers not reported.
Schawkat et al. [10]	Myrian1 (Intrasense)	ROI size: not reported<br>Location: one at the resection margin, and one at the pancreatic tail. Non-tumourous pancreatic tissue located distal to tumour, avoiding pancreatic duct and peripancreatic fat).	Single reader assessment.
Yang et al. [11]	PACS with MRI workstations	ROI size: 40-60mm^2<br>Location: the head, body and tail of the pancreas. 6 ROIs from 3 different layers per section. Mean for each ROI was recorded. Avoided large blood vessels, main pancreatic duct and peripancreatic fat.<br>FF = F / (W + F)	Two readers, blinded.
Yang et al. [12]	Siemens Sygno-imaging workstation	ROI size: about 100mm^2<br>Location: the head, body and tail of the pancreas.<br>FF = (IP - OP) / 2IP	Single reader assessment.
Yoon et al. [13]	Not reported	ROI size: mean area 208.6mm^2 ± 124.9 (SD)<br>Location: near resection margin in pancreatic parenchyma.	Single reader assessment.

Abbreviations: FF, fat fraction; ROI, region of interest; IP, in-phase (signal); OP, out-of-phase (signal); CV, coefficient of variation; F, fat (signal); W, water (signal); IDEAL, Iterative Decomposition of water and fat with Echo Asymmetry and Least-squares estimation; CSE-MRI, chemical shift-encoded magnetic resonance imaging; MRS, magnetic resonance spectroscopy; PACS, picture archiving and communication system; MRI, magnetic resonance imaging.

Supplementary Table 4. Processing and measurement methods of histology studies.

Study ID	No. of readers, blinding	Site of pancreatic sampling	Stain and processing	FF measurement and calculation	Histology measured FF, %	CSE-MRI measured FF, %
Coe et al. [1]	1, U	Resection margin	H&E, digital images were taken	x10 magnification images were greyscaled on Adobe Photoshop, then contrast was brought out between grey parenchyma and white fat. Magic wand tool used to highlight areas of fat. Histogram tool used. $FF = \frac{pixels\ highlighted\ by\ wand}{total\ pixels}$ Mean FF derived from 8 slides.	2.2 (0.3–9.3) *	3.6 (0.1–10.4) *
Fukui et al. [2]	1, Y	Non-tumorous tissue at the resection stump	H&E, three areas of slides were analysed (FOV = 5.4 mm x 7.2 mm)	ImageJ version 1.51; light microscopy. $FF = \frac{intralobular + interlobular\ fat}{total\ pancreatic\ parenchyma}$	0–40 **	0–38 **
Kiemen et al. [4]	3, Y	Not reported	H&E, formalin-fixed; neural network was used to rapidly label tissue microanatomy	Extra-pancreatic fat was excluded by three readers; mean taken as FF value. $FF = \frac{pixels\ showing\ fat}{total\ pixels}$	10.0 (13.7) †	5.3 (12.0) †
Schawkat et al. [10]	2, U	Resection margin & pancreas tail	H&E	Intra-pancreatic fat was graded as L1 = 0% to 10% deposition, L2 = 11% to 30%, and L3 = greater than 30%.	Not reported	11.1 (8) ‡
Yoon et al. [13]	1, Y	Resection margin	H&E	$FF = \frac{area\ of\ intraparenchymal\ fat}{total\ area\ of\ pancreatic\ parenchyma}$, as visualised on the slide.	5 (0–70; 1–10) §	6.5 (1.7–39.1; 4.6–10.8) §

Abbreviations: Y, yes; N, no; U, unclear; H&E, haematoxylin and eosin; FF, fat fraction; CSE-MRI, chemical shift-encoded magnetic resonance imaging.

Footnotes: * Median (range); ** approximate range; † median (interquartile range); ‡ mean (SD) for pancreas tail region; § median (range; 25th-75th quartile).

References

1. Coe PO, Williams SR, Morris DM et al (2018) Development of MR quantified pancreatic fat deposition as a cancer risk biomarker. Pancreatology 18:429–437
2. Fukui H, Hori M, Fukuda Y et al (2019) Evaluation of fatty pancreas by proton density fat fraction using 3-T magnetic resonance imaging and its association with pancreatic cancer. Eur J Radiol 118:25–31
3. Hu HH, Kim H, Nayak KS, Goran MI (2010) Comparison of fat–water MRI and single-voxel MRS in the assessment of hepatic and pancreatic fat fractions in humans. Obesity 18:841–847
4. Kiemen AL, Dbouk M, Diwan EA et al (2024). Magnetic resonance imaging-based assessment of pancreatic fat strongly correlates with histology-based assessment of pancreas composition. Pancreas 53:e180–e186
5. Kořínek R, Gajdošík M, Trattnig S, Starčuk Z, Krššák M (2020) Low-level fat fraction quantification at 3 T: comparative study of different tools for water–fat reconstruction and MR spectroscopy. Magn Reson Mater Phys 33:455–468
6. Kühn J, Berthold F, Mayerle J et al (2015) Pancreatic steatosis demonstrated at MR imaging in the general population: clinical relevance. Radiology 276:129–136
7. Li J, Xie Y, Yuan F, Song B, Tang C (2011) Noninvasive quantification of pancreatic fat in healthy male population using chemical shift magnetic resonance imaging: effect of aging on pancreatic fat content. Pancreas 40:295–299
8. Machann J, Hasenbalg M, Dienes J et al (2022) Short-term variability of proton density fat fraction in pancreas and liver assessed by multiecho chemical-shift encoding-based MRI at 3 T. J Magn Reson Imaging 56:1018–1026
9. Moquillaza EH, Weiss K, Steinhelfer L et al (2025) Whole pancreas water T1 mapping at 3 tesla. Magn Reson Mater Phys 38:271–283
10. Schawkat K, Eshmuminov D, Lenggenhager D et al (2018) Preoperative evaluation of pancreatic fibrosis and lipomatosis: correlation of magnetic resonance findings with histology using magnetization transfer imaging and multigradient echo magnetic resonance imaging. Invest Radiol 53:720–727
11. Yang JZ, Murphy R, Lu J (2022) A fat fraction phantom for establishing new convolutional neural network to determine the pancreatic fat deposition. Heliyon 8:e12478
12. Yang W, Xie Y, Song B, Xia C, Tang C, Li J (2019) Effects of aging and menopause on pancreatic fat fraction in healthy women population: A strobe-compliant article. Med (Baltimore) 98:e14451
13. Yoon JH, Lee JM, Lee KB et al (2016) Pancreatic steatosis and fibrosis: quantitative assessment with preoperative multiparametric MR imaging. Radiology 279:140–150
